# Repair of Partial and Transitional Atrioventricular Septal Defects: Mid-term Outcomes in a Single-center Cohort

**DOI:** 10.31083/RCM44492

**Published:** 2026-01-16

**Authors:** Zewen Chen, Yifan Li, Wen Xie, Tianyu Chen, Hailong Qiu, Xiaohua Li, Xiaobing Liu, Jimei Chen, Yong Zhang, Shusheng Wen

**Affiliations:** ^1^Department of Cardiac Surgery, Guangdong Cardiovascular Institute, Guangdong Provincial People's Hospital (Guangdong Academy of Medical Sciences), Southern Medical University, 510080 Guangzhou, Guangdong, China; ^2^Department of Pediatric Cardiology, Guangdong Cardiovascular Institute, Guangdong Provincial People's Hospital (Guangdong Academy of Medical Sciences), Southern Medical University, 510080 Guangzhou, Guangdong, China; ^3^Department of Cardiology, Inselspital, Bern University Hospital, University of Bern, 3010 Bern, Switzerland

**Keywords:** partial atrioventricular septal defect, transitional atrioventricular septal defect, surgical outcomes, atrioventricular valve regurgitation, reoperation, ring annuloplasty

## Abstract

**Background:**

Surgical repair of partial and transitional atrioventricular septal defects (AVSDs) aims to achieve optimal outcomes with minimal need for reintervention. This study aimed to evaluate the mid-term outcomes of AVSD repair in both pediatric and adult populations.

**Methods:**

We retrospectively reviewed all patients who underwent surgical repair for partial or transitional AVSDs at our center between January 2019 and December 2022. Key outcomes, including mortality, reoperation, and atrioventricular valve (AVV) repair strategies, were assessed during follow-up.

**Results:**

A total of 136 patients were included (partial AVSD, n = 100; transitional AVSD, n = 36), with a median follow-up of 50.5 months. The median hospital stay was 14 days. No early or late deaths occurred. Reoperation was required in four patients (2.9%); all reoperations included left atrioventricular valve (LAVV) reoperation. However, reoperation rates did not differ significantly between AVSD subtypes (*p* = 1.000) or age groups (*p* = 0.177). The incidence of moderate or greater LAVV regurgitation showed no significant difference between patients with and without ring annuloplasty, either postoperatively or at the final follow-up (both *p* = 1.000).

**Conclusions:**

Surgical repair of partial and transitional AVSDs results in excellent mid-term survival and a low reoperation rate across both pediatric and adult patients. Continued refinement of AVV repair strategies remains essential to reduce the risk of LAVV reintervention and prevent left ventricular outflow tract obstruction. Long-term follow-up is warranted to improve the evaluation of the durability of techniques such as suture annuloplasty and ring annuloplasty.

## 1. Introduction

Atrioventricular septal defects (AVSDs) represent 4–7% of congenital heart defects (CHDs) and are frequently associated with Down syndrome [1]. Based on the degree of incomplete septal development surrounding the atrioventricular valve (AVV), AVSD was delineated into complete, partial, and transitional types [2]. Among these, nearly one-third of patients present with partial AVSD (21.5%) or transitional AVSD (11.8%) [3]. Over the past few decades, advances in techniques and perioperative management have contributed to substantial improvements in surgical outcomes for these patients [4, 5].

Despite significant progress, most research has focused separately on pediatric and adult populations, with limited comprehensive studies covering the entire age spectrum. Interestingly, in China, the proportion of adult AVSD patients is relatively higher compared to developed countries [6], largely due to socio-economic factors and delayed access to medical intervention. This unique demographic distribution may influence surgical outcomes, highlighting the need for tailored strategies. To address this gap, we conducted a single-center retrospective analysis of patients with partial and transitional AVSD who underwent surgical repair at our institution. The primary objectives of this study were to evaluate the surgical outcomes in patients with partial and transitional AVSD and assess an age-dependent strategy for AVV repair. The overarching goal of this study was to determine the optimal surgical techniques for the effective repair of partial and transitional AVSD across different age groups.

## 2. Materials and Methods

### 2.1 Study Population

This was a single-center, retrospective study conducted at Guangdong Provincial People’s Hospital, analyzing all patients with partial and transitional AVSD who underwent primary surgical repair between January 2019 and December 2022. Patients with concomitant complex congenital heart defects, such as tetralogy of Fallot, Ebstein anomaly, or other major structural anomalies, were excluded from the analysis. In addition, patients whose primary repair was performed at outside institutions were not included. For patients with more than moderate pulmonary hypertension (PH) identified preoperatively by echocardiography, right heart catheterization was performed. Those with a pulmonary vascular resistance (PVR) greater than 5 Wood units (WU) were excluded from this study. Patients were stratified into four age groups according to age at the time of surgery: infants (<1 year), toddlers (≥1 year to <3 years), children (≥3 years to <18 years), or adults (≥18 years).

This study was approved by the Ethics Review Board of Guangdong Provincial People’s Hospital. A waiver of informed consent was granted due to the retrospective design. The follow-up period was defined as the time from the date of surgery to the most recent clinical evaluation by a cardiac surgeon at our institution.

### 2.2 Data Collection and Definitions

Patient data were retrospectively obtained from institutional medical records. Transthoracic echocardiography findings were reviewed at four time points: preoperatively, postoperatively, at the most recent follow-up, and prior to any reintervention. The severity of AVV regurgitation was assessed using color Doppler imaging and graded according to jet area into four categories: none/trivial, mild, moderate, or severe. Left ventricular outflow tract (LVOT) gradients were calculated using Bernoulli’s simplified equation [7]. A maximum instantaneous gradient of ≥20 mmHg was considered indicative of clinically significant LVOT obstruction (LVOTO) [6].

### 2.3 Surgical Technique

Closure of the primum atrial septal defect (ASD) was performed using either autologous or bovine pericardial patches. In the setting of a transitional AVSD, the restrictive ventricular septal defect (VSD) was closed using the modified single-patch technique. The zone of apposition of the left atrioventricular valve (LAVV) was routinely closed in all patients unless there was a concern for potential postoperative LAVV stenosis.

Additional valvuloplasty procedures were tailored according to the patient’s age and the severity of AVV regurgitation. When ring annuloplasty was indicated, an incomplete C-shaped ring was used for both LAVV and right atrioventricular valve (RAVV) repairs to preserve native leaflet motion and accommodate annular dynamics. Intraoperative transesophageal echocardiography was performed following right atrial closure to assess valve competence. Residual AVV regurgitation of trace or mild degree was considered acceptable for achieving satisfactory surgical outcomes.

### 2.4 Statistical Analysis

Continuous variables are reported as medians (ranges), and comparisons between groups were performed using the Student’s *t*-test or the Wilcoxon rank-sum test, as appropriate based on data distribution. Categorical variables are summarized as frequency (percentage), and group comparisons were conducted using the Chi-squared test or Fisher’s exact test, depending on expected cell counts.

All statistical analyses were performed using IBM SPSS statistics 29.0.1.0 (SPSS Inc., Armonk, NY, USA) on macOS Sequoia. Figures were created using GraphPad Prism 10.4.0 (GraphPad Software, LLC., San Diego, CA, USA). A two-tailed *p*-value < 0.05 was considered statistically significant.

## 3. Results

Between January 2019 and December 2022, a total of 136 patients underwent surgical repair for AVSD at our center, including 100 patients (73.5%) with partial AVSD and 36 patients (26.5%) with transitional AVSD (Table 1). Among them, 92 patients (67.6%) were female. The age at the time of surgery ranged from 1 month to 64 years, with a median age of 22 years. The cohort included a relatively balanced distribution of pediatric and adult patients, comprising 15 infants (11.0%), 14 toddlers (10.2%), 33 children (24.3%) and 74 adults (54.4%). Median body weight was 45.0 kg, ranging from 3.4 to 92.0 kg. Only one patient, diagnosed with partial AVSD, had Down syndrome. Two patients (1.5%) were identified with significant PH, including 1 patient (1.0%) in the partial AVSD subgroup. None of the patients had clinically significant LVOTO prior to surgical intervention.

**Table 1.** **Baseline demographics**.

Variable		Partial AVSD N = 100	Transitional AVSD N = 36	*p* value	Total N = 136
Age		24 y (1 m to 61 y)	16 y (1 m to 64 y)	0.620	22 y (1 m to 64 y)
Weight (kg)		46.0 (3.4–92.0)	43.0 (4.0–88.0)	0.556	45.0 (3.4–92.0)
Female		66 (66.0%)	26 (72.2%)	0.494	92 (67.6%)
Infants		9 (9.0%)	6 (16.7%)	0.343	15 (11.0%)
Toddlers		11 (11.0%)	3 (8.3%)	0.895	14 (10.2%)
Children		23 (23.0%)	10 (27.8%)	0.566	33 (24.3%)
Adults		57 (57.0%)	17 (47.2%)	0.312	74 (54.4%)
Down syndrome		1 (1.0%)	0 (0%)	1.000	1 (0.7%)
Significant PH		1 (1.0%)	1(2.7%)	0.461	2 (1.5%)
Significant LVOTO		0	0		0
Preoperative LAVV regurgitation moderate or more		75 (75.0%)	24 (66.7%)	0.335	99 (72.8%)
	Infant	7 (7.0%)	4 (11.1%)	0.534	11 (8.1%)
	Toddlers	10 (10.0%)	1 (2.8%)	0.384	11 (8.1%)
	Children	15 (15.0%)	8 (22.2%)	0.178	23 (16.9%)
	Adult	43 (43.0%)	11 (30.6%)	0.325	54 (39.7%)
Preoperative RAVV regurgitation moderate or more		56 (56.0%)	23 (63.9%)	0.411	79 (58.1%)
	Infant	6 (6.0%)	4 (11.1%)	0.661	10 (7.4%)
	Toddlers	5 (5.0%)	1 (2.8%)	0.666	6 (4.4%)
	Children	9 (9.0%)	6 (16.7%)	0.474	15 (11.0%)
	Adult	36 (36.0%)	12 (33.3%)	0.317	48 (35.3%)

AVSD, atrioventricular septal defect; PH, pulmonary hypertension; LVOTO, left ventricular outflow tract obstruction; LAVV, left atrioventricular valve; RAVV, right atrioventricular valve; m, month; y, year.

Preoperative echocardiographic assessment confirmed that all patients had LAVV regurgitation. Among them, 99 patients (72.8%) were classified as having moderate or severe regurgitation. No statistically significant differences were observed in LAVV regurgitation between the partial and transitional AVSD subgroups (*p* = 0.335). Stratified analysis showed that 45 pediatric patients (33.1%) and 54 adult patients (39.7%) had moderate or severe LAVV regurgitation, with no significant differences (*p* = 0.959). Regarding RAVV regurgitation, 79 patients (58.1%) demonstrated moderate or severe RAVV regurgitation preoperatively, including 31 pediatric patients (22.8%) and 48 adult patients (35.3%). Similar to the findings for LAVV regurgitation, there were no significant differences in RAVV regurgitation between AVSD subtypes (*p* = 0.441) or between different age categories (*p* = 0.080).

### 3.1 Intraoperative Details

Most operations were performed via standard median sternotomy under cardiopulmonary bypass (CPB), with the exception of seven thoracoscopic procedures in adult patients (5.1%), one minimally invasive procedure through a lower sternotomy in a 9-month-old infant (0.7%), and one subaxillary incision approach in a 6-year-old child (0.7%). All patients underwent pericardial patch closure of primum ASD using either an autologous or bovine pericardial patch. In cases of transitional AVSD, restricted VSD were repaired using a modified single-patch technique.

The zone of apposition of the LAVV was closed with interrupted polypropylene sutures in all patients. LAVV suture annuloplasty was performed in 45 patients (33.1%), and annuloplasty rings were utilized in 30 patients (22.1%). One patient required LAVV replacement during the initial operation due to an unsatisfactory outcome after valvuloplasty. For the RAVV, suture annuloplasty was performed in 57 patients (41.9%), and annuloplasty rings were used in 45 patients (33.1%). Detailed information on valvuloplasty techniques, CPB and aortic cross-clamp (AXC) times by AVSD subtypes is presented in Table 2.

**Table 2.** **Operative demographics**.

Variable		Partial AVSD N = 100	Transitional AVSD N = 36	*p* value	Total N = 136
CPB time (min)		110 (55–324)	124 (66–350)	0.373	119 (55–350)
AXC time (min)		68 (29–214)	77 (41–175)	0.401	71 (29–214)
Second bypass run		2 (2.0%)	1 (2.8%)	1.000	3 (2.2%)
Zone of apposition of the LAVV closure		100 (100%)	36 (100%)		136 (100%)
LAVV suture annuloplasty		29 (29.0%)	16 (44.4%)	0.091	45 (33.1%)
LAVV ring annuloplasty		25 (25.0%)	5 (13.9%)	0.168	30 (22.1%)
RAVV suture annuloplasty		39 (39.0%)	18 (50.0%)	0.251	57 (41.9%)
RAVV ring annuloplasty		35 (35.0%)	10 (27.8%)	0.430	45 (33.1%)
Concomitant correction		15 (15.0%)	1 (2.7%)	0.069	16 (11.8%)
	Single atrium	10 (10.0%)	1 (2.7%)	0.287	11 (8.1%)
	ASVC	3 (3.0%)	0	0.565	3 (2.2%)
	RVOTO	2 (2.0%)	0	1.000	2 (1.5%)
	Cor triatriatum	1 (1.0%)	0	1.000	1 (0.7%)
	Coronary artery fistula	1 (1.0%)	0	1.000	1 (0.7%)
Delayed closure		0	0		0

CPB, cardiopulmonary bypass; AXC, aortic cross-clamp; ASVC, anomalous systemic venous connection; RVOTO, right ventricular outflow tract obstruction.

Sixteen patients (11.8%) underwent concomitant surgical correction for cardiac anomalies other than closure of patent foramen ovale (PFO) or patent ductus arteriosus (PDA). These included 11 patients (8.1%) who underwent single atrium repair, 3 patients (2.2%) with correction of anomalous systemic venous connection (ASVC), 2 patients (1.5%) who underwent resection of right ventricular outflow tract obstruction (RVOTO), 1 patient (0.7%) with cor triatriatum resection, and 1 patient (0.7%) with repair of a coronary artery fistula.

A second bypass run was required in two patients (1.4%): one due to persistent LAVV regurgitation following initial repair and the other due to intraoperative detection of LVOTO. Both conditions were successfully addressed during the second bypass. None of the patients required delayed closure.

### 3.2 Postoperative Outcomes

The median hospital stay was 14 days (range, 6–62 days) and there was no in-hospital mortality. None of the patient required extracorporeal membrane oxygenation (ECMO) following primary repair of partial or transitional AVSD, or during their intensive care unit (ICU) stay.

Postoperative surgical outcomes and complications are summarized in Table 3. Moderate or severe LAVV regurgitation was observed in 10 patients (7.4%), including 7 patients (7.0%) in the partial AVSD group and 3 patients (8.3%) in the transitional AVSD group, with no statistically significant difference between groups (*p* = 1.000). When stratified by age, moderate or severe LAVV regurgitation occurred in 5 pediatric patients (8.1%) and 5 adult patients (6.8%), also without a significant difference (*p* = 1.000). Moderate or severe RAVV regurgitation was found in 9 patients (6.6%), including 5 patients (5.0%) in the partial AVSD group and 4 (11.1%) in the transitional AVSD group. This difference was not statistically significant (*p* = 0.382).

**Table 3.** **Postoperative outcomes and complications**.

Variable		Partial AVSD N = 100	Transitional AVSD N = 36	*p* value	Total N = 136
Postoperative LAVV regurgitation moderate or more		7 (7.0%)	3 (8.3%)	1.000	10 (7.4%)
	Infant	1 (1.0%)	2 (5.6%)	0.183	3 (2.2%)
	Toddlers	1 (1.0%)	0	1.000	1 (0.7%)
	Children	1 (1.0%)	0	1.000	1 (0.7%)
	Adult	4 (4.0%)	1 (2.8%)	1.000	5 (3.7%)
Postoperative RAVV regurgitation moderate or more		5 (5.0%)	4 (11.1%)	0.382	9 (6.6%)
	Infant	0	2 (5.6%)	0.167	2 (1.5%)
	Toddlers	1 (1.0%)	0	1.000	1 (0.7%)
	Children	1 (1.0%)	1 (2.8%)	1.000	2 (1.5%)
	Adult	3 (3.0%)	1 (2.8%)	0.524	4 (2.9%)
Postoperative significant LVOTO		0	0		0
Residual ASD/VSD		0	0		0
Perioperative reoperation		3 (3.0%)	1 (2.8%)	1.000	4 (2.9%)
Permanent pacemaker		1 (1.0%)	1 (2.8%)	0.461	2 (1.5%)
ECMO		0	0		0
Infection		5 (5.0%)	3 (8.3%)	0.436	8 (5.9%)
Pericardial effusion		2 (2.0%)	0	1.000	2 (1.5%)
Chylothorax		0	0		0
In-hospital mortality		0	0		0
LOS (d)		13 (6–62)	14 (6–31)	0.480	14 (6–62)

ASD, atrial septal defect; VSD, ventricular septal defect; ECMO, extracorporeal membrane oxygenation; LOS, length of stay.

During the postoperative period, reoperations were required in 4 patients (2.9%). Indications included severe LAVV regurgitation with a small annulus in two patients and third-degree atrioventricular blocks in the other two. The median time from primary repair to postoperative reoperation was 15 days (range, 15–21 days). At discharge, echocardiography revealed no cases of severe LVOTO or LAVV stenosis. None of the patients had residual ASD or VSD.

### 3.3 Follow-up Outcomes

The median follow-up was 50.5 months (range, 15 days to 79 months). There was no late mortality across all age groups, with overall survival remaining 100%. Moreover, there was also no mortality after reoperation (Table 4).

**Table 4.** **Follow-up characteristics**.

Variable		Partial AVSD N = 100	Transitional AVSD N = 36	*p* value	Total N = 136
Most recent follow-up LAVV regurgitation moderate or more		10 (10.0%)	4 (11.1%)	1.000	14 (10.3%)
	Infant	1 (1.0%)	3 (8.3%)	0.041	4 (2.9%)
	Toddlers	2 (2.0%)	0	1.000	2 (1.5%)
	Children	1 (1.0%)	0	1.000	1 (0.7%)
	Adult	6 (6.0%)	1 (2.8%)	0.559	6 (4.4%)
Most recent follow-up RAVV regurgitation moderate or more		4 (4.0%)	1 (2.8%)	1.000	5 (3.7%)
	Infant	1 (1.0%)	0	1.000	3 (2.2%)
	Toddlers	0	0		0
	Children	1 (1.0%)	0	1.000	1 (0.7%)
	Adult	2 (2.0%)	1 (2.8%)	1.000	3 (2.2%)
Most recent follow-up significant LVOTO		0	0		0
Late mortality		0	0		0
Reoperation		3 (3.0%)	1 (2.8%)	1.000	4 (2.9%)

**Fig. 1.**
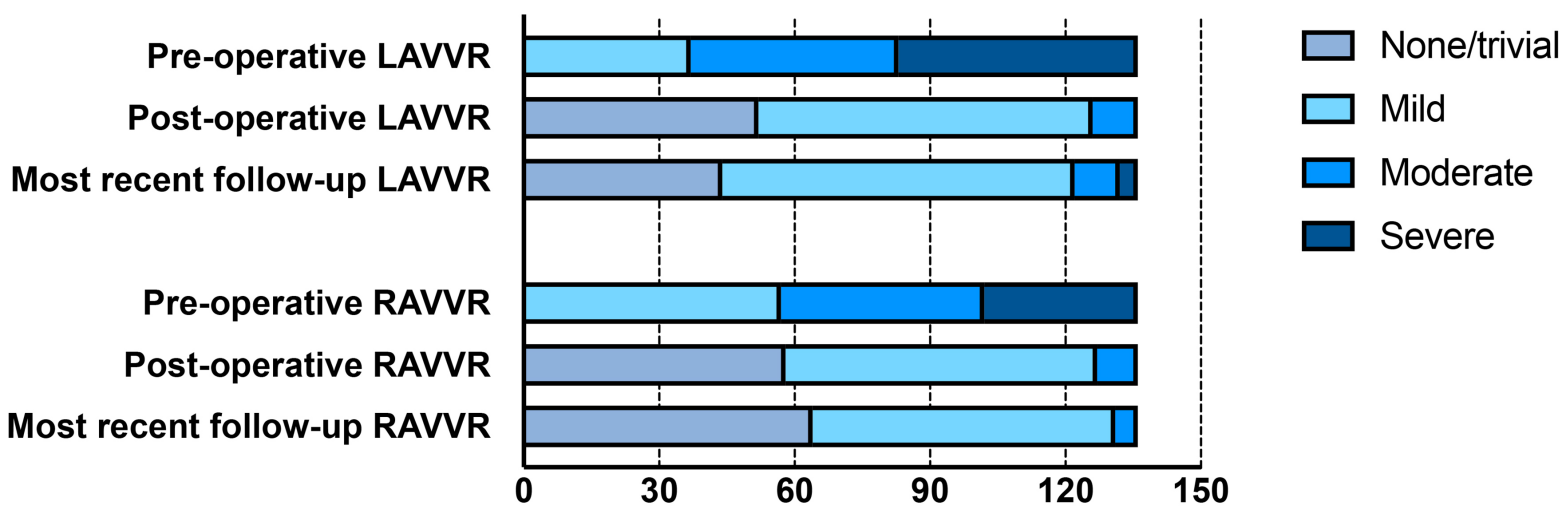
**Valve regurgitation at pre-operation, post-operation, and most recent follow-up**. LAVVR, left atrioventricular valve regurgitation; RAVVR, right atrioventricular valve regurgitation.

At the latest follow-up echocardiography, moderate or severe LAVV regurgitation was observed in 14 patients (10.3%), including 10 patients (10.0%) in the partial AVSD group and 4 patients (11.1%) in the transitional AVSD group. This difference was not statistically significant (*p* = 1.000). When stratified by age, 7 pediatric patients (11.3%) and 7 adult patients (9.5%) demonstrated moderate or severe LAVV regurgitation, also without a significant intergroup difference (*p* = 0.726). Moderate or severe RAVV regurgitation was identified in 5 patients (3.7%), with 4 patients (4.0%) in the partial AVSD group and 1 patient (2.8%) in the transitional AVSD group showing no significant difference between groups (*p* = 1.000). The distribution of individuals with LAVV and RAVV regurgitation is shown in Fig. 1. None of the patients exhibited severe LVOTO or LAVV stenosis during follow-up.

**Fig. 2.**
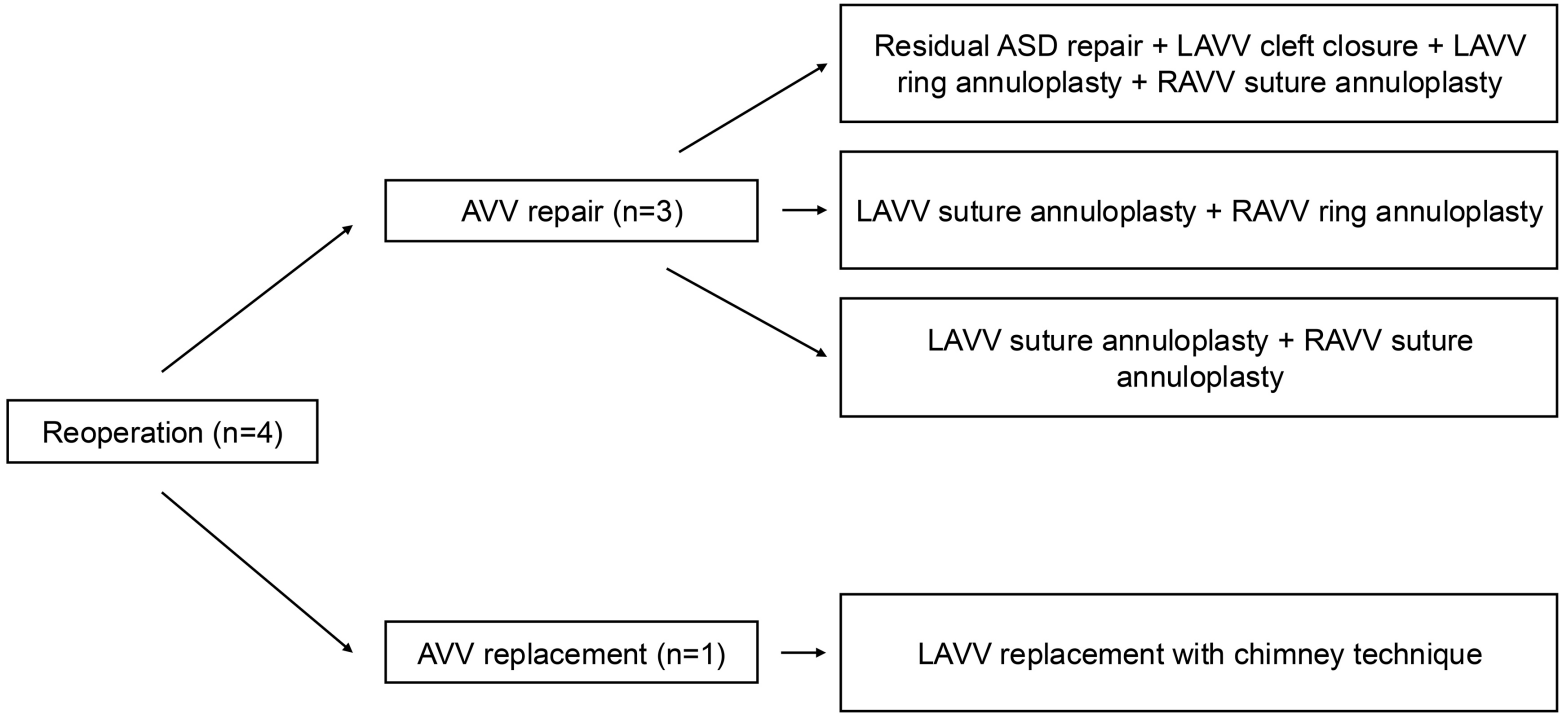
**Reoperation procedures**. AVV, atrioventricular valve.

**Fig. 3.**
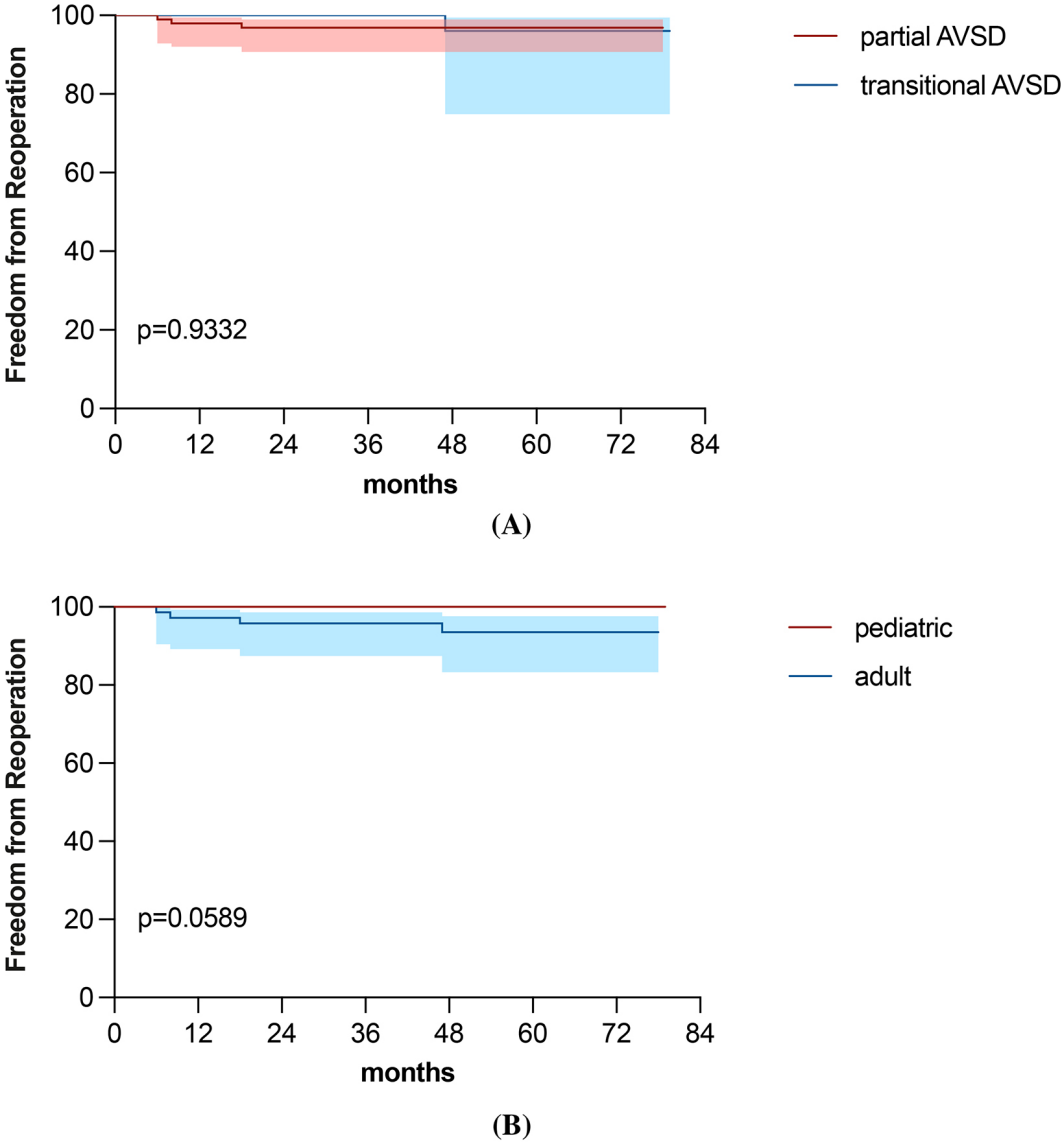
**Freedom from reoperation by AVSD subtypes (A) and age categories (B)**.

During follow-up, four patients (2.9%) required reoperation, with no cases necessitating more than one reoperation. The median interval between primary repair and reoperation was 13 months (range, 6–47 months). The individual procedures included LAVV repair in three cases (37.5%), LAVV replacement in one case (12.5%), RAVV repair in three cases (37.5%) and closure of a residual ASD in one case (12.5%). The reoperation procedures are shown in Fig. 2. Notably, all reoperations included LAVV reoperation. The freedom from reoperation at 1, 3, and 5 years following initial repair was 98.5%, 97.7% and 96.6%, respectively (Fig. 3A). When stratified by age group, freedom from reoperation in adult patients was 97.2%, 95.8%, and 93.5% at 1, 3, and 5 years, respectively (Fig. 3B). No reoperations were required in the pediatric cohort, and the difference between age groups was not statistically significant (*p* = 0.177).

## 4. Discussion

We present a cohort study from a single center analyzing the mid-term surgical outcomes for patients with partial and transitional AVSD. Consistent with previous reports on prevalence of CHDs in China [8], our study demonstrated a higher proportion of female patients in both adult and pediatric groups. Compared with previous studies, our population included a higher proportion of patients with moderate or greater preoperative LAVV or RAVV regurgitation in both pediatric and adult groups [6, 9]. At our center, the primary indications for early surgical intervention in such patients are severe AVV regurgitation or symptomatic congestive heart failure. Overall, operative and postoperative outcomes were excellent, with a 100% survival rate, underscoring the efficacy of contemporary surgical techniques and perioperative care.

Mortality associated with the surgical repair of partial and transitional AVSD has been consistently low in previous literature. In 2000, El-Najdawi *et al*. [10] reported an early mortality of 2% and a 5-year mortality of 6%. In 2015, a 37-year retrospective study by Buratto *et al*. [5] documented a 1.2% operative mortality, with 10- and 30-year mortality of 4% and 6%, respectively. In 2019, Mery *et al*. [6] reported perioperative mortality of 0.8% and a 10-year mortality of 2% in a cohort of 265 patients from Texas Children’s Hospital. More recently, Cai *et al*. [11] reported no in-hospital mortality following transitional AVSD repair and a 1.8% mortality in partial AVSD repair from Children’s Hospital of Fudan University in 2022. Our findings compare favorably, with no early or late mortality observed, likely reflecting continued advancements in surgical technique and perioperative care.

The overall reoperation rate in our cohort was 2.9% (3.0% for partial AVSD and 2.8% for transitional AVSD), which is slightly lower than that reported in several recent large single-center series [9, 12]. Most reoperations occurred within the first year following initial repair, with cumulative reoperation rates of 1.5% at 12 months and 36 months, and 2.9% at 60 months. The 96.6% freedom from reoperation at 5 years is consistent with published data [4, 11, 13].

LAVV reintervention remains the most common indication for reoperation in AVSD patients, with reported rates ranging from 3% to 13% in adults with partial or transitional AVSD [5, 12, 14]. In our cohort, all reoperations involved LAVV dysfunction and were exclusively observed in adult patients. No pediatric patients required reoperation. In contrast to other studies where LVOTO is frequently cited as a second common indication for reoperation [4, 15, 16], we observed no LVOTO-related reoperations in pediatric patients postoperatively or during follow-up. Only one adult patient developed LVOTO intraoperatively, which was resolved during a second bypass run. Notably, no patients required permanent pacemaker (PPM) implantation during follow-up. The only PPM implantations occurred as early postoperative interventions in two adult patients (1.5%), following third-degree atrioventricular block after primary surgery during the same hospital admission.

The LAVV cleft has been recognized as a key factor in postoperative LAVV regurgitation and subsequent LAVV reoperation [4, 17]. A previous study has shown that the presence of a residual cleft at discharge, regardless of its severity, is associated with a significantly increased risk of late LAVV reintervention [13]. At our center, all LAVV clefts were routinely closed using interrupted, nonpledgeted polypropylene sutures. This technique is designed to minimize the risk of both residual regurgitation and cleft dehiscence. Compared to continuous suturing, which is technically more straightforward but more vulnerable to failure if a single point gives way, the interrupted approach offers greater durability and precision. While pledgeted sutures may offer added reinforcement, they can limit leaflet mobility and pose a risk for calcification over time. In our cohort, no reoperations were attributed to residual or reopened LAVV clefts, supporting the effectiveness and reliability of our cleft repair strategy.

In the surgical correction of AVSD, a variety of LAVV repair techniques—such as ring annuloplasty, Kay’s annuloplasty, and other tailored approaches—are commonly employed [18]. In our cohort, pediatric patients, particularly those with larger body sizes, underwent posterior suture annuloplasty when significant LAVV regurgitation persisted after cleft closure. Ring annuloplasty was selectively applied in older children and adolescents presenting with notable annular dilatation (ages: 13, 13, 15 and 17; weights: 44 kg, 49 kg, 63 kg and 71 kg, respectively). Excessive intervention in younger children was avoided due to the potential risk of LVOTO during growth. Prior studies have highlighted the efficacy of annuloplasty in addressing LAVV regurgitation, with a preference for conservative strategies over prosthetic materials to preserve valve dynamics and reduce long-term complications [19, 20]. In adult patients, cleft closure—alone or combined with posterior suture annuloplasty—served as the first-line approach. Ring annuloplasty was utilized when patients exhibited both severe regurgitation and significant annular enlargement. When analyzing the outcomes separately for LAVV ring annuloplasty, there was no significant difference in the incidence of moderate or severe LAVV regurgitation between patients with and without annuloplasty rings during postoperative (*p* = 1.000) and follow-up (*p* = 1.000) evaluations (Table 5). This individualized, anatomy- and age-dependent strategy resulted in favorable valve outcomes across both pediatric and adult populations, with no statistically significant differences between age groups. Notably, the 5-year freedom from LAVV reoperation in pediatric patients at our center was 100%, which compares favorably with recent reports from Turkey (91%) [9] and markedly surpasses a 34% reintervention rate reported in a U.S. series over medium-term follow-up [17]. In addition to its clinical effectiveness, this tailored approach also contributed to cost reduction, which is particularly meaningful for patients with limited financial resources. However, these results are based on mid-term outcomes, and longer follow-up will be necessary to further evaluate the durability of ring annuloplasty and other surgical strategies.

**Table 5.** **Annuloplasty ring analysis for LAVV regurgitation**.

Variable	LAVV annuloplasty ring		*p* value	Total N = 136
	Without	With		
Post-operative LAVV regurgitation less than moderate	98	28	1.000	126
Post-operative LAVV regurgitation moderate or more	8	2		10
Most recent follow-up LAVV regurgitation less than moderate	95	27	1.000	122
Most recent follow-up LAVV regurgitation moderate or more	11	3		14

### Limitation

It is important to note that echocardiographic assessments, including the grading of AVV regurgitation, are operator-dependent. Variations in results may arise due to differences in the experience and technique of the professionals performing the echocardiograms. While efforts were made to standardize echocardiographic evaluations within our institution, this limitation should be considered when interpreting the findings.

## 5. Conclusions

Surgical repair of partial and transitional AVSD can achieve excellent outcomes with minimal mortality and reoperation incidence. Nonetheless, the AVV repair strategy requires careful consideration to mitigate potential LAVV reintervention and LVOTO following primary correction.

## Availability of Data and Materials

All data reported in this paper will also be shared by the lead contact upon request.
